# Changes in Sperm Motility and Capacitation Induce Chromosomal Aberration of the Bovine Embryo following Intracytoplasmic Sperm Injection

**DOI:** 10.1371/journal.pone.0129285

**Published:** 2015-06-10

**Authors:** Yoku Kato, Yoshikazu Nagao

**Affiliations:** 1 Department of Animal Production Science, United Graduate School of Agricultural Science, Tokyo University of Agriculture and Technology, Fuchu, 183-8509, Japan; 2 University Farm, Faculty of Agriculture, Utsunomiya University, Mohka, Tochigi 321-4415, Japan; USA, UNITED STATES

## Abstract

Intracytoplasmic sperm injection (ICSI) has become the method of choice to treat human male infertility. One of the outstanding problems associated with this technique is our current lack of knowledge concerning the effect of sperm capacitation and motility upon the subsequent development of oocytes following ICSI. In the present study, we first examined the capacitation state of sperm exhibiting normal motility, along with sperm that had been activated, and examined the effect of reactive oxygen species (ROS) produced by these sperm types upon embryogenesis following bovine *in vitro* fertilization (IVF) and ICSI. Data showed that activated sperm reduced the chromosomal integrity of IVF/ICSI embryos at the blastocyst stage, while capacitated sperm produced ROS in capacitation media. Secondly, we treated sperm with carbonyl cyanide m-chlorophenyl hydrazine (CCCP), a chemical known to uncouple cell respiration within the mitochondria, and investigated the effect of this treatment upon blastocyst formation and chromosomal integrity at the blastocyst stage. Activated sperm in which the mitochondria had been treated with CCCP reduced levels of chromosomal aberration at the blastocyst stage following ICSI, by reducing mitochondrial activity in activated sperm. In conclusion, these findings suggest that capacitated sperm exhibiting activated motility induced chromosomal aberration during development to the blastocyst stage following ICSI. The injection of sperm exhibiting normal motility, or activated sperm in which mitochondrial activity had been reduced, improved the quality of ICSI-derived embryos. Therefore, the selection of sperm exhibiting progressive motility may not always be better for early embryo development and fetal growth following human ICSI, and that the use of a bovine model may contribute to a deeper understanding of sperm selection for human ICSI embryo development.

## Introduction

Intracytoplasmic sperm injection (ICSI) has become the method of choice to treat human male infertility [1]. ICSI has been successfully used to produce live offspring in many species of animals [2–4], and has also been introduced into the treatment of human infertility [5]. Since its first introduction, ICSI has resulted in the birth of over 2.5 million children [6]. Sperm capacitation is a mechanism that is necessary for sperm to be incorporated into the oocyte during both *in vivo* and *in vitro* fertilization. However, ICSI bypasses the need for sperm capacitation, acrosome reaction, along with gamete binding and fusion, by artificially injecting a single sperm directly into a metaphase II oocyte. Interestingly, some recent reports recommend induction of the acrosome reaction prior to injecting sperm for ICSI as this leads to good embryogenesis and successful implantation [7–9]. Other reports claim that sperm capacitation is not necessary for ICSI and could in fact, reduce embryo development [10, 11].

Mammalian capacitation has previously been correlated with changes in sperm plasma membrane fluidity, intracellular ion concentration, reactive oxygen species (ROS), metabolism and motility [12,13]. Capacitating sperm are also known to produce controlled amounts of ROS [14]. We previously demonstrated that capacitated bovine sperm exhibited activated motility in capacitation medium [15]. These reports highlight the possibility that capacitated sperm exhibiting activated motility could be associated with the production of ROS by the plasma membrane and mitochondria [14,16]. On the other hand, increased ROS generation from sperm would inevitably lead to oxidative stress, including sperm DNA fragmentation and reduced motility [17,18]. ROS derived from oxygen within the culture environment is also known to impair the development of *in vitro* matured and fertilized bovine oocytes [19,20]. Capacitating mouse sperm has been shown to affect embryo development and the chromosomal integrity of embryos during *in vitro* culture following mouse ICSI [10]. In mouse sperm, capacitation also increased the levels of asynchronous remodeling and resulted in delayed pronuclei formation and DNA synthesis following sperm injection [11]. Moreover, rhesus macaque embryos arising from ICSI carried out with sperm exhibiting ROS damage, resulted in impairments to mitosis, DNA integrity, and development to the blastocyst stage *in vitro* [21].

However, one of the outstanding problems associated with ICSI is our current lack of knowledge concerning the effect of sperm capacitation, motility, and mitochondrial status upon blastocyst formation and chromosomal integrity following ICSI. In the present study, we carried out a systematic analysis of these important sperm parameters with regard to embryo development following ICSI. In order to accomplish this, we examined sperm capacitation, motility, mitochondrial activity and ROS production, upon the development of embryos created by ICSI with sperm exhibiting inactivated/activated motility and hyper-activation.

## Materials & Methods

### Oocyte collection and *in vitro* maturation

The cattle, from which we obtained their ovaries, had been slaughtered for the public edible meat. Those ovaries were discarded without any utilization. Hence, an ethics statement in our paper was not required to use ovary for experiments as previously described [22–24]. Bovine ovaries were obtained from an abattoir, placed in saline containing a 0.1% solution of antibiotic/antimycotic (Gibco Laboratories, Grand Island, NY, USA: AB), and transported to the laboratory within 1–3 h. Oocyte collection and *in vitro* maturation of oocytes were carried out as previously described [25]. More than 30 ovaries from Japanese black and holstein cows were collected to permit appropriate an experimental replication. Oocytes with intact cumulus cells and evenly granulated cytoplasm were selected, washed and cultured in modified TCM-199 (m-TCM199) for 22–25 h at 39.0°C under 5% CO_2_ in air with high humidity. m-TCM199 consisted of hepes-buffered medium 199 (Gibco) supplemented with 0.1% (w/v) polyvinyl alcohol (PVA;Sigma Chemical Co, St. Louis,MO.), 0.5 mM sodium pyruvate (Nacalai Tesque Inc., Kyoto, Japan), 1% AB, 0.02 AU/ml FSH (Antrin, Denka), and 1 μg/ml estradiol-17β (Sigma). The rates of *in vitro* maturation after 22–24 hrs were more than 95% in all experiments.

### Washing, pre-incubation of sperm and insemination

Sperm washing and pre-incubation was carried out as previously described [25]. The motility ratios (%) of frozen-thawed semen were greater than 70% before sperm washing with percoll. Fresh semen were frozen at Genetics Hokkaido Co., Ltd. In brief, frozen-thawed sperm were washed with a discontinuous percoll solution. Washed sperm (2.0 x 10^8^ cells/ml) were incubated in Brackett and Oliphant medium (BO) supplemented with 0.05% (w/v) PVA, 1% AB (BO·PVA: non-capacitation media: control) for 0, 2 and 4 hrs at 39.0°C under 5% CO_2_ in air with high humidity. Washed sperm were then cultured in BO·PVA with methyl-β-cyclodextrin (MBCD: BO·MBCD: capacitation media) for 0, 2 and 4 h. Following culture in capacitation media for 4 hrs, sperm were introduced into BO supplemented with progesterone (P) or carbonyl cyanide m-chlorophenyl hydrazone (CCCP), a chemical known to uncouple cell respiration within the mitochondria for 0.5–1 hour. Matured cumulus-oocyte complex (COCs) or denuded oocytes (10 oocytes/100 μL droplet) were inseminated in the presence of the sperm (2.0 ×10^6^ cells/ml) which were activated immediately after pre-incubation for 2-4hrs. Oocytes and spermatozoa were co-incubated at 39.0°C under 5% CO_2_ in air with high humidity.

### Analysis of sperm motility

**Fig 1:**
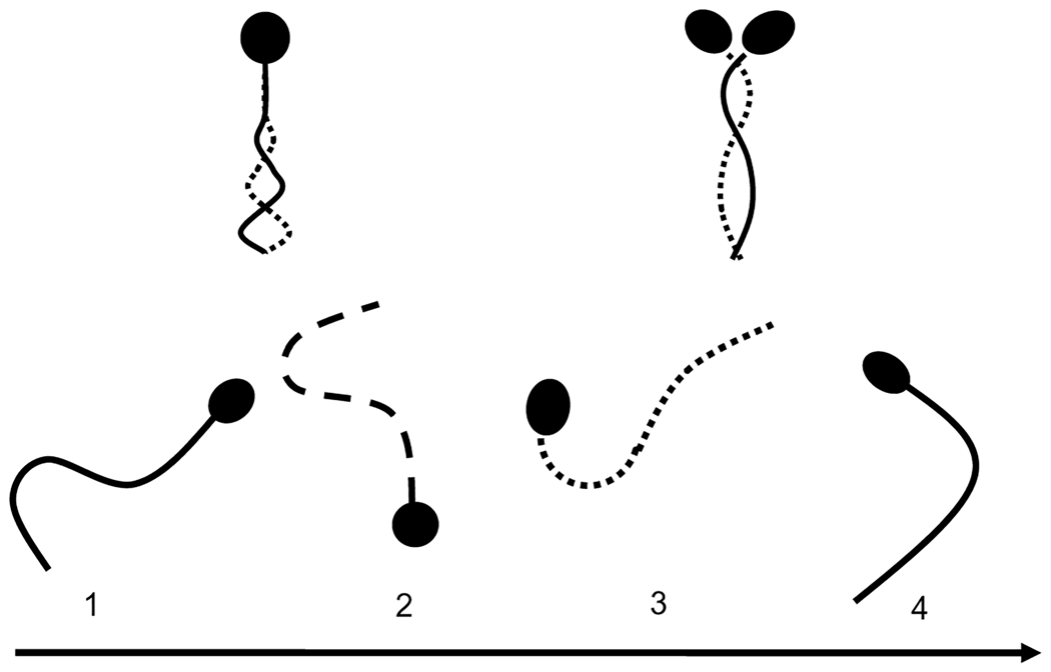
Sperm motility patterns of bovine spermatozoa cultured in non-capacitation media and capacitation media. Top left; normal motile sperm, right; sperm exhibiting activated motility, bottom; hyper-activated motility.

Sperm motility was categorized according to motility patterns as previously described [15, 26]. Over 100 sperm were examined in PBS containing 10% polyvinylpyrrolidone (PVP) solution in order to reduce and consider sperm motility on an interference contrast microscope. The ratios (%) of normal motility, activated, and hyper-activated motility sperm were determined as follows. Normal motile sperm exhibited a motile tail (Fig 1, Top left). Following the activation of motility, sperm head and flagella beat in an almost symmetrical fashion, propelling the sperm in almost linear trajectories [27–29] (Fig 1, Top right). Hyper-activated motility was characterized by asymmetrical beating of the flagellum resulting in a circular or vigorous ‘‘figure of 8” trajectory [27–29] (Fig 1, below). The proportion (%) of motile sperm refers to the number of motile sperm divided by the number of total sperm, including all motile and immotile sperm in each media, respectively. The proportion (%) of activated sperm refers to the number of such sperm divided by the number of motile sperm in each media, respectively. Finally, the proportion (%) of hyper-activated motility sperm refers to the number of such sperm divided by the number of motile sperm in each media, respectively.

### Normal, activated and hyper-activated motility of individual sperm and the chlortetracycline fluorescence assay

**Fig 2:**
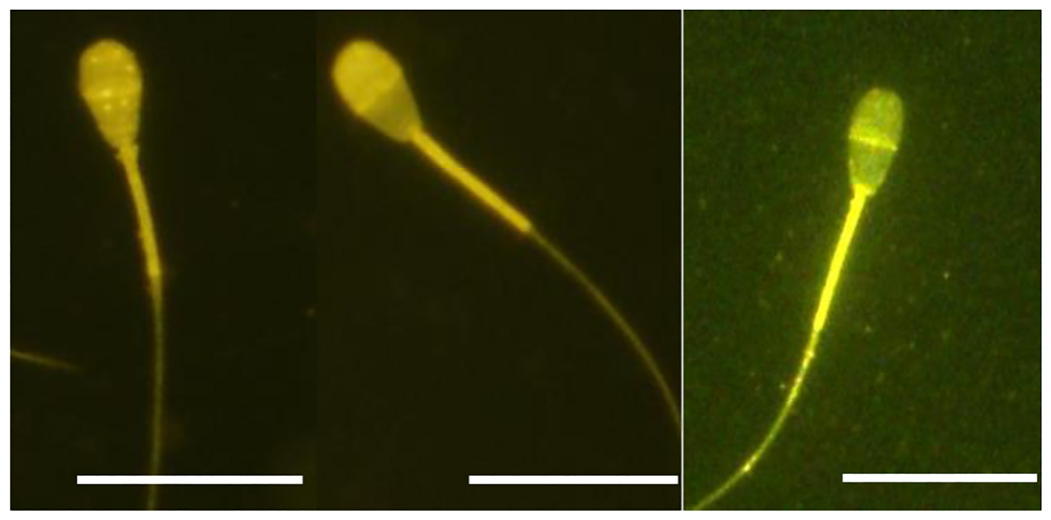
Sperm patterns arising from the chlortetracycline fluorescence assay. Left; F (uncapacitated)-pattern, central; B (capacitated)-pattern, right; AR (acrosome-reacted)-pattern. Bovine normal motile and activated sperm were incubated in the three types of media and analysed by CTC staining. Bar = 20 μm (B).

Single sperm were collected and examined after pre-incubation in capacitation media or non-capacitation media. Single sperm were collected using an injection pipette immediately prior to CTC staining as previously described [15]. Ten to twenty sperm exhibiting normal motility, activated or hyper-activated motility were collected in PBS containing 10% PVP. Sperm were placed into non-capacitation media on a glass slide (Matsunami, Japan). The chlortetracycline (CTC) stain was used to evaluate capacitation status as previously described [15, 30]. Precipitated sperm were mixed thoroughly with 750 μM CTC solution (Sigma Chemical Co.), 5 mM cysteine (Sigma Chemical Co.), 130 mM NaCl (Kanto Chemical, Japan), 20 mM Tris (Sigma, pH 7.8), fixed with 12.5% (w/v) paraformaldehyde in 0.5 M Tris-HCl (pH 7.4), and finally placed on a glass slide and covered with a coverslip. Sperm were assessed for CTC-staining using a 400 to 440-nm filter and a DM 455 dichroic mirror fitted to a fluorescent microscope. CTC-staining patterns were classified into three types according to the method described by Fraser et al [30]: F (uncapacitated)-pattern, bright fluorescence detected over the whole region of the sperm head; B (capacitated)-pattern, fluorescence detected on the sperm head but not over the post-acrosomal region; and AR (acrosome-reacted)-pattern, weak fluorescence observed over the sperm head with a bright band sometimes present in the equatorial region (Fig 2).

### Immunolabeling of tyrosine phosphorylation

Tyrosine phosphorylation was carried out in bovine sperm by immunofluorescence using 4G10 (4G10: Millipore corporation. Billerica, MA) and anti-mouse IgG conjugated with phycoerythrin (EI-2000: Vector Labratory,Inc. Burlingame, CA) [31]. For immunofluorescence microscopy, washed sperm were stained in suspension, dried onto poly-lysine-coated glass slides and then fixed. Bovine sperm were fixed in PBS containing 2% paraformaldehyde for 30 min at room temperature (RT). Sperm were then washed once in PBS and blocked with PBS containing 0.1% bovine serum albumin (BSA: PBS·BSA) for 30 min before the addition of primary antibodies. The monoclonal antibody 4G10 was used at a dilution of 1:100 in PBS·BSA. Incubations were performed for 60 min at RT with three washes in PBS·BSA between incubations. The sperm pellet was then incubated with anti-mouse IgG conjugated with phycoerythrin (diluted by 1:100) in PBS supplemented with 3 mg/ml BSA for 1h at RT. The sperm suspension was then centrifuged at 300×g for 3 min. After removal of the supernatant, the sperm were washed twice, as described above, to remove unbound secondary antibody. Samples were then mounted, kept for up to one day at 4°C in the dark, and finally examined by fluorescent microscopy (Olympus).

### Evaluation of individual sperm mitochondria by a JC-1 probe

**Fig 3:**
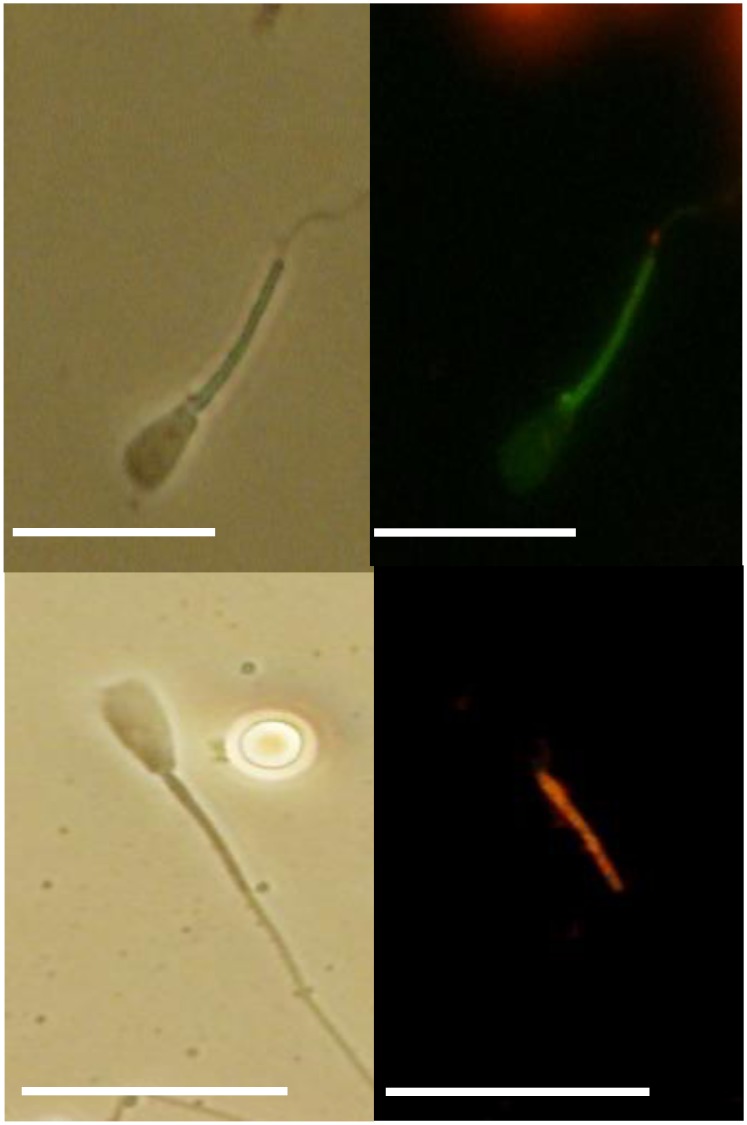
Mitochondrial activity of sperm by JC-1 staining. Top; Sperm annotated with green staining represent inactive mitochondria with no membrane potential (left: differential interference contrast: DIC, right: JC-1), Bottom; sperm annotated with bright orange staining represent active mitochondria with high membrane potential (left: DIC, right: JC-1). Bar = 20 μm.

To evaluate changes in mitochondrial membrane potential, sperm were labeled with potential-sensitive 5,5’,6,6’-tetrachloro-1,1’,3,3’-tetraethylbenzimidazolylcarbocyanine iodide (JC-1; Molecular Probes, Eugene, OR) for 10 min [32]. Live mitochondria were labeled in PVP solution supplemented with JC-1 for 10 min at 39.0°C. Sperm exhibiting normal motility or activated motility were collected in PBS containing 10% PVP on a glass slide within 5 minutes as previously described [15]. Those sperm in PVP solution were placed onto a glass slide, fixed in PBS containing 2% paraformaldehyde for 30 min at RT and washed three times in PBS·BSA. After that, sperm were covered with PBS·BSA and a cover slip. Samples were kept for up to one day at 4°C in the dark and finally examined by fluorescent microscopy. The percentage of sperm with green (inactive mitochondria, no membrane potential, Fig 3B, above), bright orange or faint orange (active mitochondria, high membrane potential Fig 3B, below) were evaluated visually by fluorescence microscopy.

### Intracytoplasmic sperm injection and embryo culture

Intracytoplasmic sperm injection was performed as described previously [33] with some modifications. Briefly, after maturation, cumulus cells were thoroughly dispersed with 0.1% hyaluronidase (Sigma) and removed by vortexing. After removal of the cumulus cells, only oocytes with a visible first polar body were selected for experimental use. Selected oocytes were washed three times in m-TCM199 without FSH and estradiol-17β. Before sperm injection, motile sperm were immobilized by applying several piezo pulses (M-701, Suruga Seiki, Shizuoka city, Japan). The immobilized spermatozoon was drawn into the injection pipette tail first and the zona pellucida drilled by applying several piezo pulses. Then, the pipette was advanced mechanically into the ooplasm. ICSI was performed after 3–4 hrs of sperm culture in capacitation media or non-capacitation media. After injection with sperm, oocytes were immediately transferred to modified synthetic oviduct fluid with BSA [34] (SOF·BSA) and cultured for 3 h. After culture, oocytes injected with sperm were treated for 3 min with 7% ethanol in TCM 199 containing 1 mg/mL PVP. Then, 15 to 50 of these ICSI embryos were placed in SOF·BSA and cultured at 39.0°C under 5% CO_2_, 5% O_2_ and 90% N_2_ at high humidity for 7–8 days [34].

### Assessment of fertilization and embryo development

Fertilization and embryo development were evaluated as previously described [25, 34]. To evaluate the pronuclear stage during fertilization, subsets of post-ICSI oocytes were fixed on a slide with 3:1 acetic acid: ethanol, and stained with 1% orcein. Oocytes considered fertilized were classified in three groups, all showing two pronuclei. Early development was examined with a stereomicroscope (×60) for cleavage and development to the blastocyst stage 7 days (168 h) after insemination. Cleaved embryos included embryos from the 2-cell stage to the blastocyst stage. Only embryos with a clear blastocoele and an inner cell mass were counted as blastocysts. In Experiment 2, the ratio (%) of blastocyst formation refers to the numbers of blastocysts divided by the number of cleaved embryos. In Experiment 3 and 4, the ratio (%) of blastocyst formation refers to the number of blastocysts divided by the number of all cultured oocytes.

### Evaluation of chromosomal integrity at the blastocyst stage

**Fig 4:**
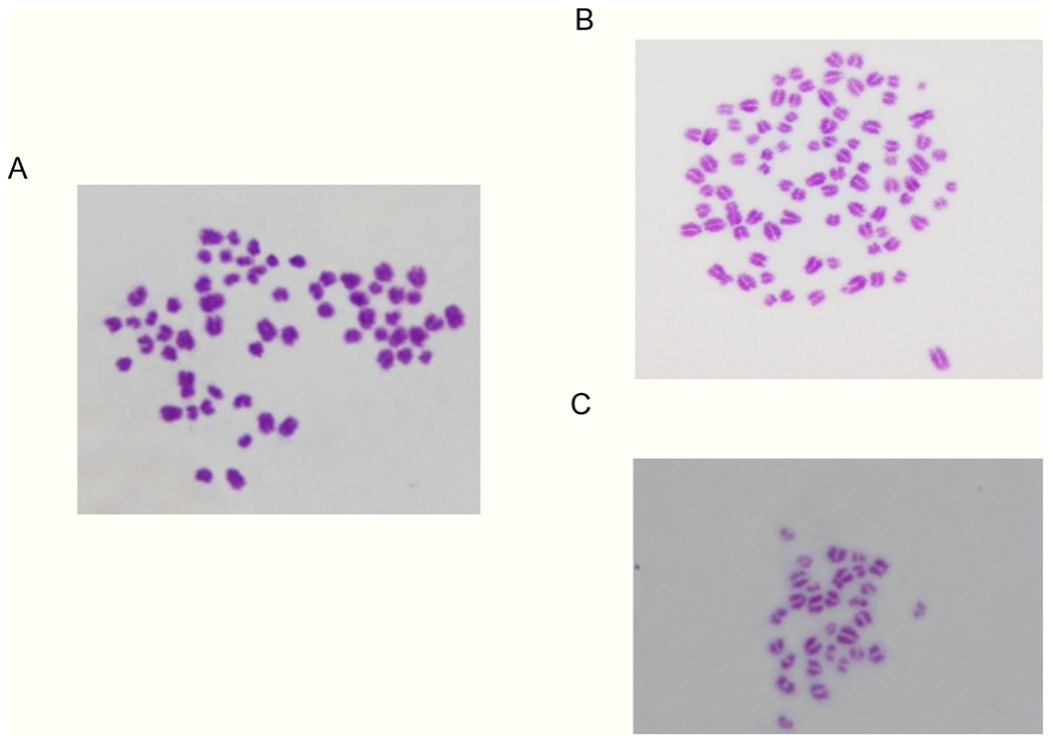
Chromosomal pattern of the bovine embryo at the blastocyst stage. (A) Typical example in which the number of chromosomes were classified as ‘normal’, 60 (= 2n). (B) Typical example in which the number of chromosomes were classified as ‘Abnormal’, 90 (= 3n). (C) Typical example in which the number of chromosomes were classified as ‘Abnormal’, 30 (= n).

Chromosomes were counted as previously described [35]. Briefly, some blastocysts were washed and placed in 1% sodium citrate solution for 15 min, and fixed by pouring 0.02 to 0.03 ml of acetic-alcohol fixative (acetic acid 1: ethanol 1) into 0.4 ml of sodium citrate solution. Blastocysts were placed onto a glass slide, immediately covered with a very small droplet of acetic acid to separate each blastomere, and then immediately re-fixed with several drops of the initial fixative. After drying completely, chromosome samples from the blastocysts were stained with 2% Giemsa solution for 10 min. The number of chromosomes were counted as ‘normal’, 60 (= 2n), or ‘abnormal’, 30 (= n) and 90 (= 3n) (Fig 4).

### Evaluation of DNA fragmentation by TUNEL staining during the pronuclear stage

**Fig 5:**
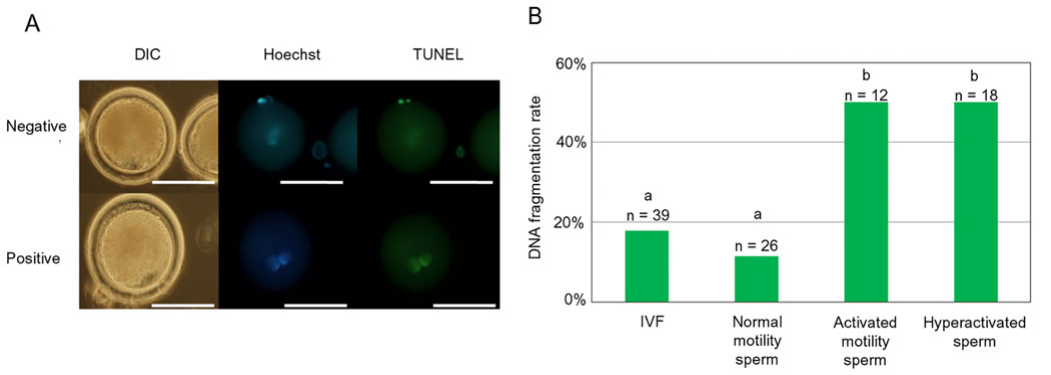
DNA fragmentation at the pronuclear stage following the injection of activated and hyper-activated sperm. (A) Positive (below) and negative (above) pattern following TUNEL staining. Differential interference contrast (DIC: left panel), Hoechst staining (central panel), and TUNEL staining (right panel). (B) Rate of DNA fragmentation in the three experimental groups. n = number of embryos. Different letters indicate significant differences as determined by the Chi Square test (P<0.01). Letters indicate significant differences (a-b). This experiment was repeated 3 times. Bar = 20 μm.

Pronuclear stage embryos were assessed by the TUNEL stain as previously described [36]. All samples were washed in PBS containing 1 mg/ml PVP (PBS·PVP), then fixed in PBS·PVP with 4% paraformaldehyde solution for 1h at RT. Afterwards, samples were rinsed with PBS·PVP and incubated in PBS·PVP including 0.5% Triton X-100 and 0.1% sodium citrate for 1 h at 39.0°C. 1 h post-incubation, TUNEL staining was performed, following the manufacturer’s instructions (Roche, Mannheim, Germany) at RT. Subsequently, PBS containing 2 mg/ml Hoechst 33342 was employed to stain samples and controls for 15 min at RT. Samples were then mounted on glass slides and examined under a fluorescent microscope as described above. The fragmented nucleus was stained yellow-green by fluorescein as a result of the TUNEL assay (Fig 5A, below). The non fragmented nucleus did not stain yellow-green (Fig 5A, above).

### Measurement of ROS produced by sperm

**Fig 6:**
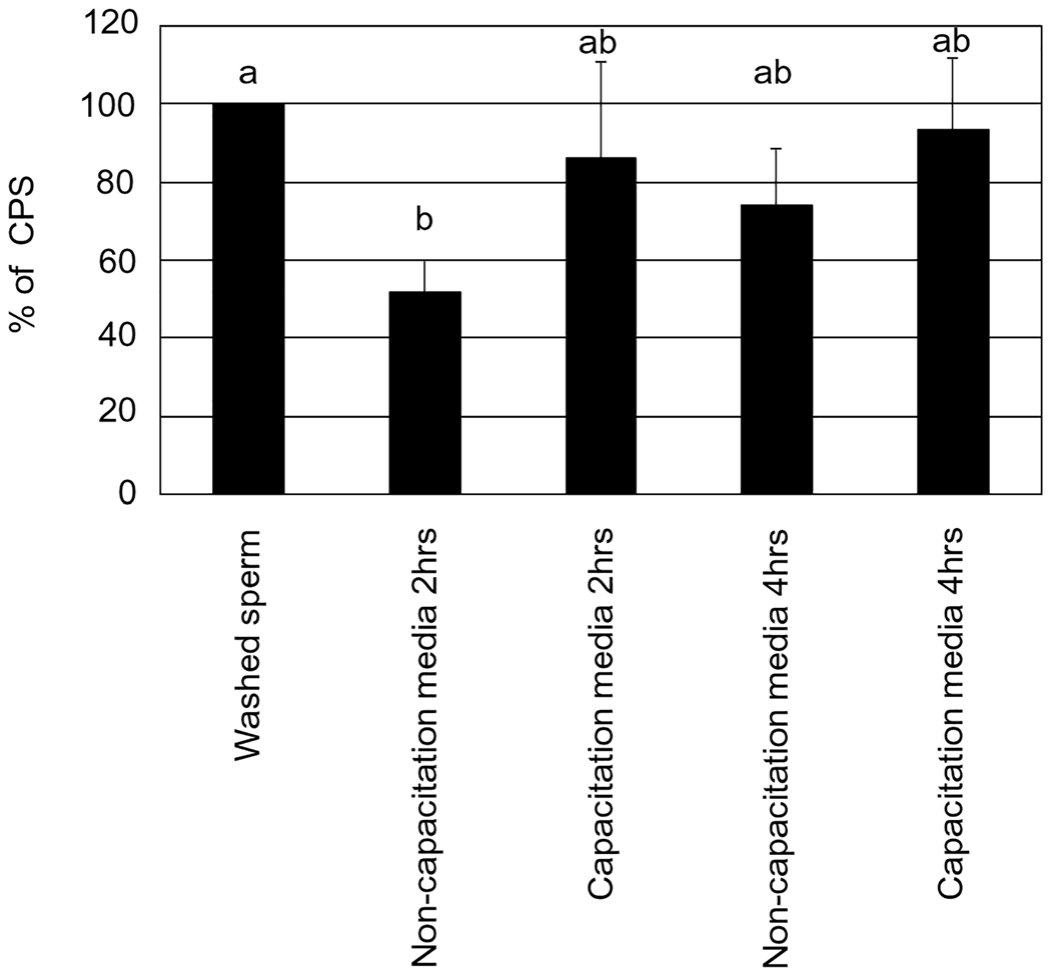
ROS production of sperm (CPS) in non-capacitation media and capacitation media. Different letters within columns indicate significant differences as determined by one-way ANOVA with Tukey-Kramer analysis (P<0.05). This experiment was repeated 3–4 times. Statistical significance compared to control: a vs b.

**Fig 7:**
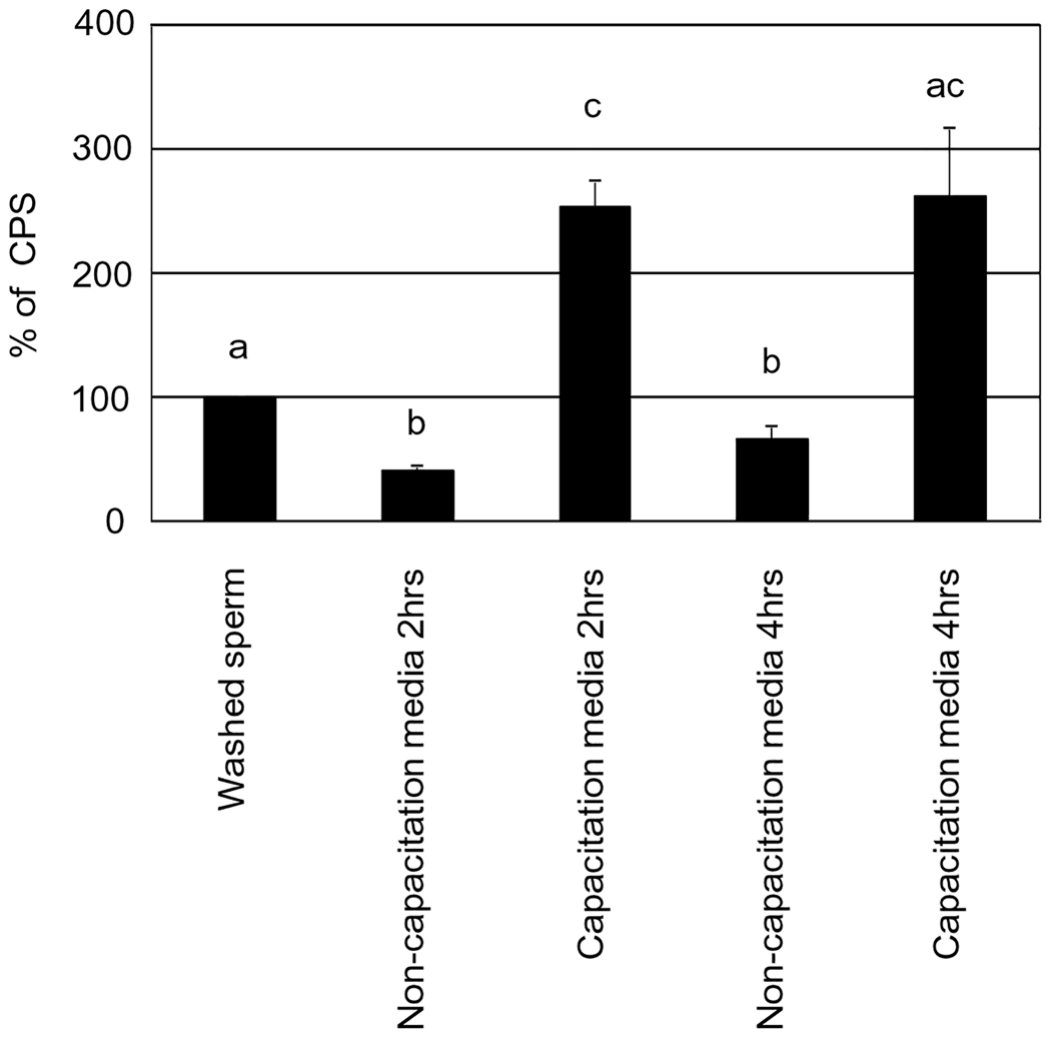
ROS production of one individual motile sperm in non-capacitation media and capacitation media. Different letters within columns indicate significant differences as determined by one-way ANOVA with Tukey-Kramer analysis (P<0.01). This experiment was repeated 3–4 times. Statistical significance compared to control: a vs b vs c.

ROS levels were estimated using the luminol assay [16]. Luminol (5-amino-2, 3-dihydro-1,4-phthalazinedione; also, 3- aminophthalic hydrazide) is used to estimate the combined levels of total ROS. The assay was sensitized by the addition of horseradish peroxidase to the sperm suspension, so as to increase the intensity of the spontaneous luminescence levels. In brief, 2 μl of 5mM luminol was added to 75μl aliquot of sperm (15 million sperm) and the reaction initiated by adding 23 μl of 2.5 units of horseradish peroxidase at RT. Results were monitored using a luminometer (Wallac 1420 VICTOR 2 T Multilabel Counter; PerkinElmer, Inc., Wellesley, MA) for 30 minutes at RT. In Fig 6, ROS production was expressed as the % of counted photons per second (CPS). In Fig 7, ROS production (CPS) was divided by the number of motile sperm in capacitation media and non-capacitation media, then ROS production by one individual sperm was expressed as a % of CPS.

### Experimental design

In experiment 1, we examined the capacitation state of normal motile, activated, and hyper-activated sperm and the ROS produced by these sperm. In experiment 2, we examined the effect of insemination with activated sperm and denuded oocytes upon embryo development. In experiment 3, we examined the effect of these sperm motile types upon blastocyst formation and chromosomal integrity at the blastocyst stage following ICSI. In experiment 4, we investigated the effect of sperm treated with CCCP, a chemical known to uncouple cell respiration within the mitochondria, upon blastocyst formation and chromosomal integrity at the blastocyst stage following ICSI.

### Statistical analyses

All experiments were replicated over 3 times. Sperm samples from at least three different bulls were used for each set of experiments. The rates of normal sperm and sperm with activated motility were evaluated for significance using one-way factorial analysis of variance (ANOVA) with Tukey-Kramer analysis. These rates were evaluated for significant differences between 0–4 hrs or between capacitation media and non-capacitation media. In Experiment 1, the means of ROS concentration, as measured by a luminometer, were evaluated between capacitation media and non-capacitation media for significance using the one-way factorial ANOVA with Tukey-Kramer analysis. In Experiment 1, 2, 3 and 4, the rates of sperm capacitation, fertilization, cleavage and blastocyst formation, along with chromosomal integrity and the rate of DNA fragmentation, were evaluated for significance using the Chi square test. In Experiment 3 and 4, the rate of fertilization, cleavage and blastocyst formation were evaluated for significant differences using the Chi square test. In all experiments, values were considered significantly different when P<0.05.

## Results

### Methyl-β-cyclodextrin (MBCD) induced the activation and capacitation of motile sperm

The proportion of activated sperm at 4 hrs was higher than that at 0 and 2 hrs in capacitation media (P<0.05, Table 1). The proportion of sperm with normal motility at 2 and 4 hrs was lower than that at 0 h in capacitation media (P<0.05, Table 1). Conversely, the proportion of activated and normal motile sperm did not change significantly with the duration of time cultured in non-capacitation media for 4 hrs (P>0.05, Table 1).

**Table 1:** The proportion (%) of sperm exhibiting normal, activated and hyper-activated motility in the two media after 0–4 hrs (n = 3–6).

Culture media		0 h	2 h	4 h
Non-capacitation media	normal motility	62.2 ± 13.1^a^	73.6 ± 5.0^a^	68.5 ± 8.4^a^
	activated motility	0.4 ± 0.4^a^	1.8 ± 0.5^a^	2.2 ± 0.85^a^
	hyper-activated motility	0	0	0
Capacitation media	normal motility	62.2 ± 13.1^a^	19.8 ± 1.5^b^	17.3 ± 2.3^b^
	activated motility	0.4 ± 0.4^a^	8.1 ± 2.2^a^	41.9 ± 4.7^b^
	hyper-activated motility	0	0	1.0 ± 0.3*

Letters within side columns indicate significant differences among 0, 2 and 4 hrs in capacitation media, respectively (a-b: one-way AVOVA with Tukey-kramer analysis: p<0.05).

**Fig 8:**
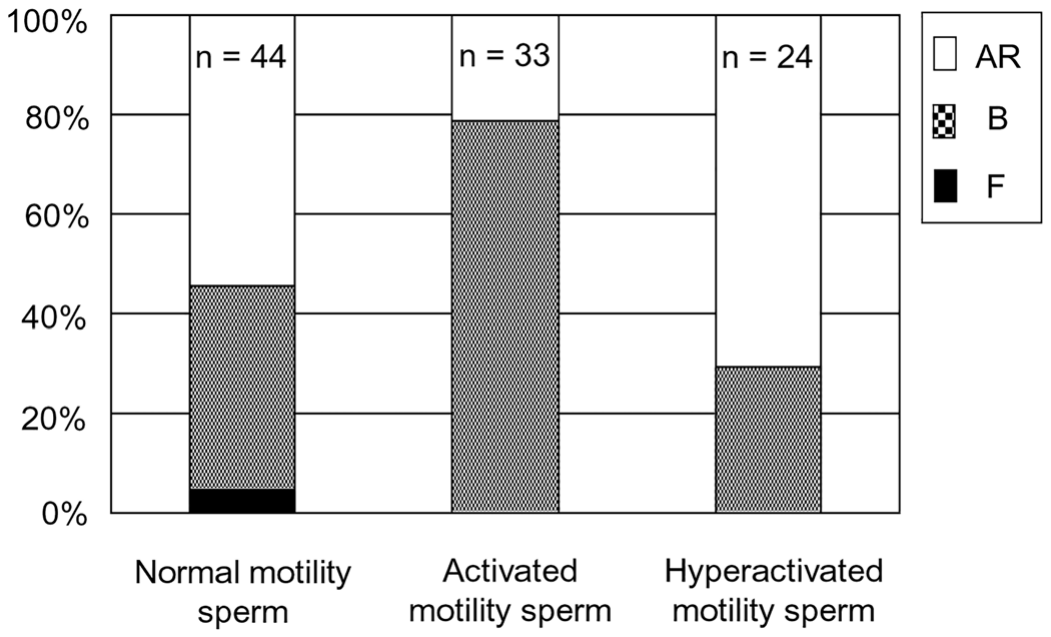
The proportion of capacitation and acrosome-reacted patterns in bovine sperm. n = number of sperm. This experiment was repeated 3 times.

In this study, the average proportion of whole live sperm displaying the B (capacitated) pattern at 0, 2 and 4 hrs of incubation were 34.2, 53.8 and 32.1% in non-capacitation media and 34.2, 71.7 and 61.6% in capacitation media, respectively. There were no differences between the present results and those reported previously [22]. The proportion of capacitation in normal motile, activated and hyper-activated sperm were 40.9 (n = 44), 78.8 (n = 33) and 29.6% (n = 24), respectively (Fig 8) after sperm were cultured for 2–4 hrs. The proportion of capacitation in activated sperm was higher than sperm exhibiting normal motility (P<0.05).

**Fig 9:**
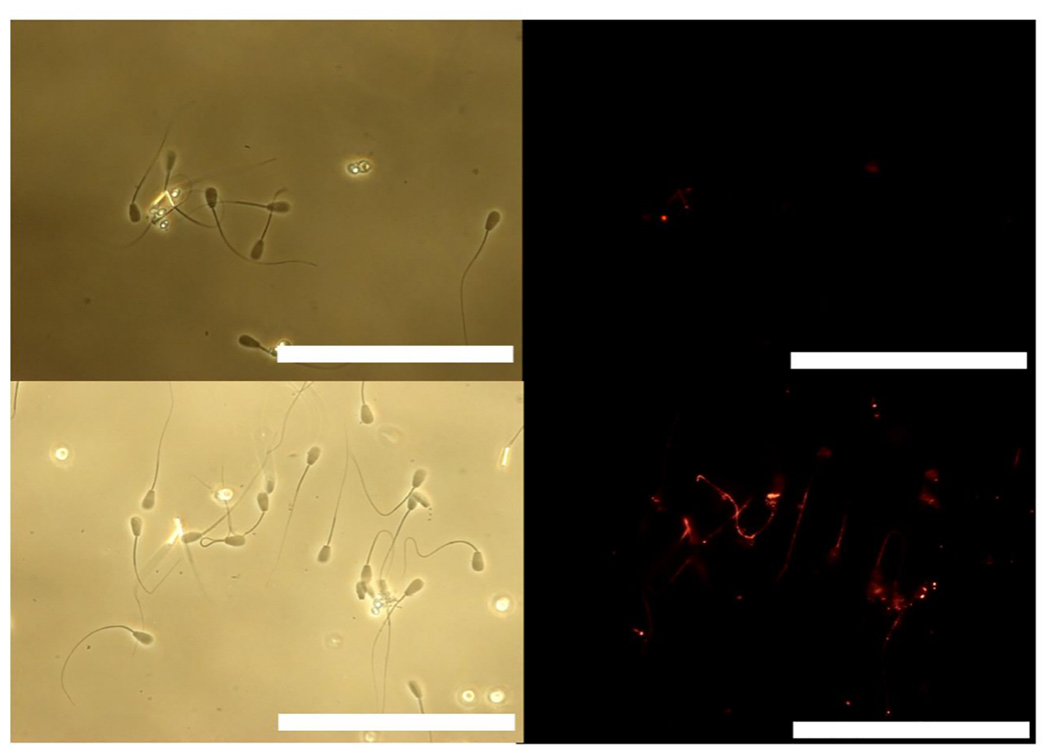
Tyrosine phosphorylation localization in sperm. Top and bottom images represent sperm cultured in non-capacitation media and capacitation media for 6hrs. Left) differential interference contrast (DIC) and right) immunofluorescence images. This experiment was repeated 3 times. Bar = 100 μm.

Sperm cultured in capacitation media exhibited a positive pattern of tyrosine phosphorylation (Fig 9). HHHowever, sperm cultured in non-capacitation media exhibited a negative pattern of tyrosine phosphorylation (Fig 9).

### Sperm generated ROS during culture

Mean values of ROS production were 141, 74 and 104 in non-capacitation media and 141, 122 and 132 CPS in capacitation media after culture for 0, 2 and 4 hrs, respectively. Following sperm culture, there was no significant difference between the values of ROS in capacitation media and non-capacitation media at 2 and 4 hrs (Fig 6, P>0.05). There was a significant reduction between 0 h and 2hrs in non-capacitation media (Fig 6, P<0.05). There was no significant reduction between 0–4 hrs in capacitation media (Fig 6, P>0.05). The number of motile sperm in capacitation media was significantly lower than that in non-capacitation media at 2 and 4 hrs (Table 1, P<0.05). Mean values of ROS production (CPS) were divided by the number of motile sperm in each media at 2 and 4 hrs. Therefore, the mean values of ROS production by one individual motile sperm in capacitation media (% of CPS) were higher than that in non-capacitation media at 2 and 4hrs (Fig 7, P<0.05).

### Blastocyst formation and chromosome integrity at the blastocyst stage following IVF of denuded oocytes with activated sperm

There were no differences in the blastocyst ratio of cleaved embryos between the two groups (Table 2, P>0.05). However, chromosomal integrity at the blastocyst stage arising from the insemination of denuded oocytes in the presence of activated sperm was lower than the insemination of COCs with activated sperm (Table 2, P<0.05).

**Table 2:** The direct effect of motile sperm in denuded oocytes on embryo developments and chromosomal aberration (n = 8).

Insemination	Total fertilization rates (%)	Normal fertilization rates (%)	No. cleaved embryos (%)	No.blastocyst (%)	Chromosomal integrity (%)
Cumulus-complex oocytes	84/116 (72.4%)^a^	62/116 (53.4%)^a^	126/229 (55.0%)^a^	33/126 (26.2%)^a^	13/23 (59.0%)^a^
Denuded oocytes	45/126 (35.7%)^a^	37/126 (29.4%)^a^	89/339 (26.3%)^a^	27/89 (30.3%)^a^	2/20 (10.0%)^b^

n = number of replicated experiments.

Letters within vertical columns indicate significant differences (a-b: Chi Square test: P<0.01).

### Blastocyst formation and chromosome integrity at the blastocyst stage following ICSI with activated sperm

There were no differences in the ratio of blastocyst formation among the four groups tested (Table 3, P>0.05). However, chromosomal integrity of the blastocyst stage arising from the injection of activated and hyper-activated sperm was lower than that with normal motile sperm and IVF (Table 3, P<0.05).

**Table 3:** The effect of type of motility upon embryo developments and chromosomal aberration in bovine embryos after ICSI (n = 8–11).

Injection sperm	Normal fertilization rates (%)	No. cleaved embryos (%)	No.blastocyst (%)	Chromosomal integrity (%)
*In vitro* fertilization	36/52 (69.2%)^a^	85/100 (85.0%)^a^	32/100 (32.0%)^a^	21/33 (63.6%)^a^
Normal motile sperm	25/34 (73.5%)^a^	74/103 (71.8%)^a^	24/103 (23.3%)^a^	18/24 (75.0%)^a^
activated sperm	30/45 (66.7%)^a^	52/78 (66.7%)^a^	19/78 (24.4%)^a^	3/13 (23.1%)^b^
hyper-activated sperm	13/15 (86.7%) ^a^	40/76 (52.6%)^a^	14/77 (18.2%)^a^	0/10 (0%)^b^

n = number of replicated experiments.

Letters within vertical columns indicate significant differences (a-b: Chi square test P<0.01).

### DNA fragmentation at the pronuclear stage following the injection of activated and hyper-activated sperm

The ratios of DNA fragmentation at the pronuclear stage following IVF and ICSI were 17.9 (n = 39) in IVF, 11.5 (n = 26) in normal motile sperm, 50.0 (n = 12) in activated motile sperm and 50.0% (n = 18) in hyper-activated sperm, respectively. The ratios following the injection of activated and hyper-activated sperm were higher than that with sperm showing normal motility and IVF (Fig 5B, P<0.05).

### The effect of CCCP treatment upon capacitated sperm

**Fig 10:**
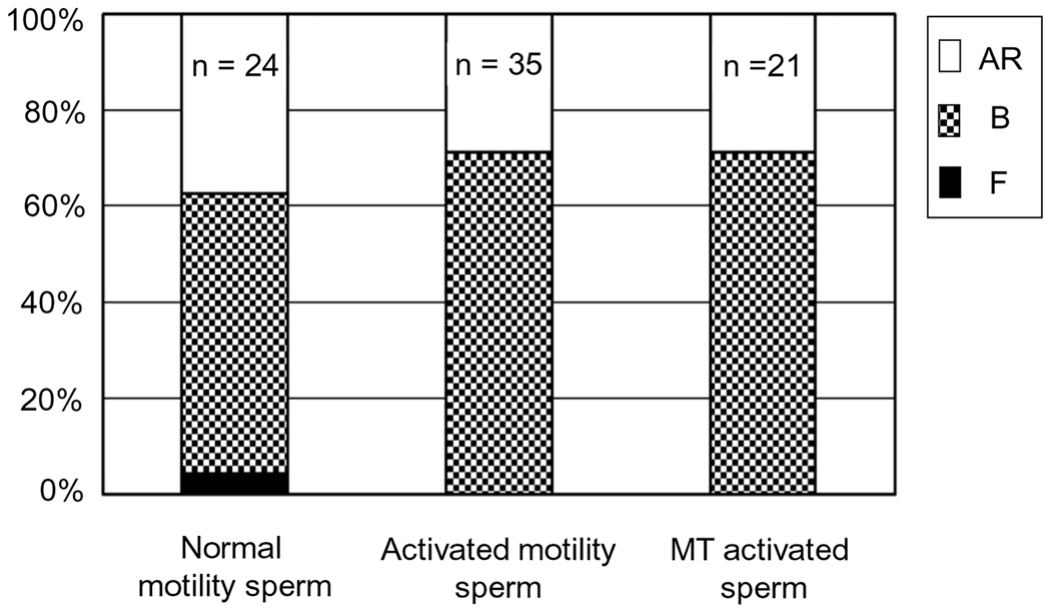
The effect of CCCP treatment upon capacitated sperm. The rate of capacitation and acrosome-reacted patterns of sperm as determined by CTC staining. This experiment was repeated 3 times.

The proportions of sperm exhibiting the characteristic pattern B of capacitation, as determined by chlortetracycline staining, were 58% (n = 24), 71% (n = 35) and 71% (n = 21) in normal motile sperm, activated sperm and mitochondria reduced activated sperm, respectively (Fig 10).

The percentage of sperm with bright orange (active mitochondria, high membrane potential, Fig 3, right-bottom) as indicated by JC-1 were 82% (n = 23), 83% (n = 37) and 33% (n = 23) in normal motile sperm, activated sperm and mitochondria reduced activated sperm, respectively. This proportion of mitochondria reduced activated sperm was lower than the other treatment groups (P<0.05)

### Effect of CCCP upon the developmental competence of sperm

There were no significant differences in the rate of embryo development to the blastocyst stage among the three groups (P>0.05, Table 4). Chromosomal integrity of the blastocyst stage of both mitochondria reduced activated sperm and normal motile sperm were higher than that of activated sperm (P<0.05, Table 4).

**Table 4:** The effect of reduced mitochondria activity upon on embryo developments and chromosomal aberration in bovine embryos after ICSI (n = 11).

Injection sperm	No. cleaved embryos (%)	No.blastocyst (%)	Chromosomal integrity (%)
Normal motile sperm	103/140 (73.5%)^a^	29/140 (20.7%)^a^	11/14 (78.6%)^a^
Activated sperm	44/80 (55.0%)^a^	20/80 (25.0%)^a^	1/14 (7.1%)^b^
mitochondria reduced activated sperm	54/86 (62.8%)^a^	27/86 (31.4%)^a^	13/15 (86.7%)^a^

n = number of replicated experiments.

Letters within vertical columns indicate significant differences in chromosomal integrity (a-b: Chi square test P<0.01).

## Discussion

ICSI has become the method of choice to treat human male infertility. One of the outstanding problems associated with this technique is our current lack of knowledge concerning the effect of sperm capacitation and motility upon the subsequent development of embryos. Most sperm used for ICSI are artificially capacitated. However, some reports claim that sperm capacitation is not necessary for ICSI and could in fact, reduce embryo development.

In experiment 1, over 30% of sperm had been activated, resulting in an increase in the production of reactive oxygen species (ROS) in capacitation media while ROS production in non-capacitation media remained steady in the presence of less than 5% of sperm which was activated. The resulting capacitation and ROS are most likely to be related to protein phosphorylation events known to occur in mammalian sperm [13]. In fact, the present study shows that capacitation in the bovine model was associated with the appearance of a tyrosine phosphoprotein complex in Experiment 1 (Fig 9). There are two reasons underlying the observed increase in ROS production. Firstly, increased production of ROS has been demonstrated from the sperm membrane during the capacitation of human and cryopreserved bovine sperm [13,14,37]. Secondly, motility causes the sperm flagellum to beat vigorously with asymmetrical beats of high amplitude [27–29]. While changes in motility would require significant ATP production by the mitochondria, these organelles can produce large levels of ROS [38,39]. Therefore, we infer that increased mitochondrial activity and increased numbers of capacitated sperm may be associated with the increased production of ROS. We further suggest that the activation of sperm contributes to the production of ROS.

In Experiment 2, the insemination of denuded oocytes with activated sperm increased the ratio of chromosomal aberration at the blastocyst stage while high levels of ROS by producing capacitated sperm was detected in Experiment 1. When sperm were incorporated into COCs during IVF, the cumulus cells protected the oocyte against oxidative stress in the media [40]. Moreover, during fertilization, sperm cells generate ROS, which not only facilitate certain sperm functions such as capacitation, hyper-activation and the acrosome reaction [41], but could also cause damage to cells [20, 42]. Therefore, we speculate that the insemination of denuded oocytes with activated sperm is related to chromosomal aberration of the blastocyst due to the destroy of the defense system of mature oocytes against ROS by the removal of cumulus cells and the increase of ROS production by capacitated sperm in capacitation media.

In Experiment 3, those embryos produced by the injection of activated and hyper-activated sperm resulted in a lower percentage of normal chromosomal integrity and DNA fragmentation while capacitated sperm could produce high levels of ROS in Experiment 1. During IVF, the sperm nucleus gradually moves away from the site of penetration and slowly becomes decondensed [43]. The decondensed sperm head, which is vulnerable to ROS damage, could move from the sperm mitochondria and the site of ROS production. During the natural fertilization process, ROS production from sperm mitochondria may be reduced drastically as a result of mitochondrial inactivation. However, during ICSI, the sperm is directly injected and deposited into the center of the oocyte [44, 45], but can still become decondensed. ROS produced by the mitochondria of sperm with activated and hyper-activated motility could thus cause damage to the paternal, and possibly maternal, genome. Moreover, the injection of sperm exhibiting ROS damage resulted in impairments to mitosis, chromosomal aberration in the 2–4 cell embryo, and development to the blastocyst stage in vitro [21]. Therefore, we suggest that detrimental effects are caused by sperm-borne ROS upon DNA fragmentation and chromosomal aberration during development to the blastocyst stage.

In Experiment 4, the injection of normal motile sperm and mitochondria reduced activated sperm did not induce chromosomal aberration at the blastocyst stage, while the uncoupling agent carbonyl cyanide m-chlorophenyl hydrazine (CCCP) reduced the mitochondrial activity of sperm and stop the mitochondrial membrane proton gradient and reduce ROS production [46]. To support the result of Experiment 4, we put forward two hypotheses. Firstly, during natural fertilization or IVF, sperm mitochondria activity would be reduced following incorporation of the sperm into the oocyte while such mitochondrial activity was not impeded in oocytes subject to ICSI. In fact, sperm mitochondria activity was regulated by progesterone produced by the cumulus cells [47]. Secondly, literature appears to suggest that the ubiquitin-dependent proteolysis of paternal mitochondria may be delayed in oocytes after ICSI and that sperm mitochondria are degraded earlier after IVF than ICSI [48], suggesting that the mitochondrial activity of sperm was not inhibited following ICSI and ROS generated by the sperm mitochondria in injected oocytes. Hence, we speculate that the reduction of mitochondrial activity of sperm exhibiting activated motility improved chromosomal integrity at the blastocyst stage following ICSI.

Interestingly, ICSI represents approximately one-half of all assisted reproductive techniques used in Europe [49]. In human ICSI, sperm exhibiting high speed motility are always selected as being the most likely to fertilize, and are thus injected into oocytes in human ICSI. However, from the view of a clinical embryologist, it was thought that the mitochondria and membrane of selected sperm are likely to be damaged by the micropipette during sperm immobilization for human ICSI. On the other hand, there is little evidence that the results of this present paper applies to humans, and a large number of healthy babies have been delivered by ICSI. Therefore, we think that our current findings are specific to bovine species and that sperm selection methodology should be investigated more thoroughly and future experiments should also assess the relationship between motility and genetic integrity, including chromosomal aberration and DNA fragmentation in mammalian species, notably mice and humans.

In conclusion, capacitated sperm showing activated and hyper-activated motility did not affect the frequency of blastocyst development, but did induce chromosomal aberration at the blastocyst stage. It is feasible that ROS produced by the sperm is a potentially dangerous factor when ICSI is carried out in humans and domestic animals. Hence, these findings suggest that the injection of sperm in which ROS production was eliminated could improve the quality of ICSI-derived bovine embryos and contribute to a deeper understanding of sperm motility and selection for human ICSI embryo development.
